# Human conditions of insulin-like growth factor-I (IGF-I) deficiency

**DOI:** 10.1186/1479-5876-10-224

**Published:** 2012-11-14

**Authors:** Juan E Puche, Inma Castilla-Cortázar

**Affiliations:** 1 Applied Molecular Medicine Institute (IMMA), School of Medicine, Department of Medical Physiology, Universidad CEU San Pablo, Madrid, Spain

**Keywords:** Laron syndrome, Liver cirrhosis, Aging, Oxidative stress, GH/IGF-I axis, Mitochondrial dysfunction, Cellular protection, Growth, Cancer protection

## Abstract

Insulin-like growth factor I (IGF-I) is a polypeptide hormone produced mainly by the liver in response to the endocrine GH stimulus, but it is also secreted by multiple tissues for autocrine/paracrine purposes. IGF-I is partly responsible for systemic GH activities although it possesses a wide number of own properties (anabolic, antioxidant, anti-inflammatory and cytoprotective actions).

IGF-I is a closely regulated hormone. Consequently, its logical therapeutical applications seems to be limited to restore physiological circulating levels in order to recover the clinical consequences of IGF-I deficiency, conditions where, despite continuous discrepancies, IGF-I treatment has never been related to oncogenesis. Currently the best characterized conditions of IGF-I deficiency are Laron Syndrome, in children; liver cirrhosis, in adults; aging including age-related-cardiovascular and neurological diseases; and more recently, intrauterine growth restriction.

The aim of this review is to summarize the increasing list of roles of IGF-I, both in physiological and pathological conditions, underlying that its potential therapeutical options seem to be limited to those proven states of local or systemic IGF-I deficiency as a replacement treatment, rather than increasing its level upper the normal range.

## Introduction

Insulin-like growth factor I (IGF-I) is a 70 aa polypeptide hormone with endocrine, paracrine, and autocrine effects. It shares >60% homology with IGF-II and by 50% homology with proinsulin structures [1].

IGFs were first described in 1957 by Salmon and Daughaday [2] when they noted that direct addition of growth hormone (GH) to costal cartilage from hypophysectomized rats *in vitro* did not significantly stimulate growth (measured by radioactive sulfate uptake). Consistently, serum from these hypophysectomized rats was also ineffective. However, normal rat serum stimulated the *in vitro* uptake of sulfate into costal cartilage from hypophysectomized rats. These results demonstrated the existence in serum of a “sulfation factor” that stimulated incorporation of ^35^Sulfate by costal cartilage.

In parallel, Froesch et al. described the non-suppressible insulin-like activity (NSILA) of two soluble serum components (NSILA I and II) by the fact that they stimulated glucose uptake into isolated rat adipocytes, sharing “insulin-like” activity, while anti-insulin antibodies were not able to abrogate their hypoglycemic effects [3].

At the same time, other investigators found some other roles for these polypeptides. Among all, their mitogenic capability, that moved them to propose the term “fraction with multiplication stimulating activity” [4].

Only when Daughaday et al. pointed out that the sulfation factors were “identical with or very similar to the smaller molecular weight component of the non-suppressible insulin-like activity” in 1972, a new nomenclature was proposed for these two molecules: somatomedin A and C, denoting substances under control and mediating the effects of GH [5].

Finally, a more extensive research on NSILAs/somatomedins carried out by Rinderknecht and Humbel [6,7] culminated with the discovery that those molecules had identical amino acid sequences to “two forms of an insulin-like hormone whose effects on cell and tissue growth predominate over those on metabolic parameters” [8]. Therefore, accordingly to their structural resemblance to proinsulin, they were finally renamed “insulin-like growth factor I and II” (IGF-I and II), molecules that also fulfill all the criteria of a somatomedin: 1) they possess insulin-like activity in the presence of insulin antibodies [3,9]; 2) they are sulfation factors [9,10]; 3) they could act as mitogens [9,11]; and 4) at least, IGF-I is growth-hormone dependent [5].

The consensus about their nomenclature [12] together with the milestone in the discovery of their amino acid sequences, which made possible the subsequent recombinant synthesis [8], opened the door to many new areas of research, and boosted the number of articles from that moment up to more than 32,500 works currently indexed in PubMed.

This historical perspective provides us a list of actions carried out by IGF-I, among others: tissue growth and development, insulin-like activity, proliferative, pro-survival/anti-aging, antioxidant, etc.

As an hormone with a wide range of physiological roles, IGF-I levels must be strictly controlled, as it has been demonstrated from *in vivo* results: six forms of high affinity IGF binding proteins (IGFBPs 1 to 6), either promoting or inhibiting IGF-I actions; a yearly increasing list of IGFBPs proteases; allelic variations and an alternative splicing are some of the mechanisms by which IGF-I is tightly maintained in a close physiological range (~286.1±52.4 ng/mL, adults between 21–40 years old) [13,14].

**Figure 1:**
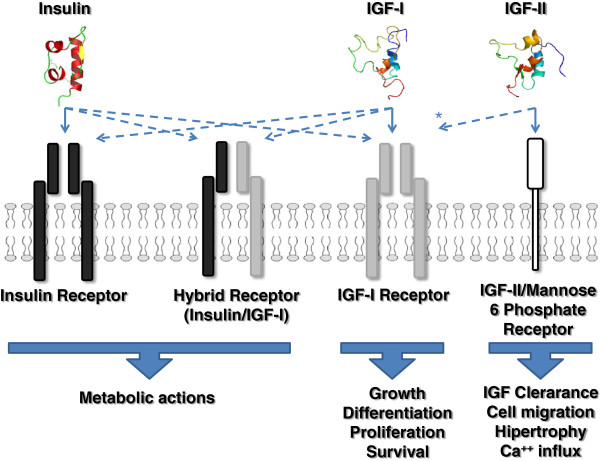
**Schematic structures of IGFs and their receptors.** Resemblances between Insulin and IGFs allow them to cross-interactions by which IGFs are able to bind to their own receptors (preferently) but also to Insulin receptor (IR) with a lower specificity. The hybrid receptor shares components from both IR and IGF-IR. * IGF-II can also interact with IGF-IR, hybrid receptor and insulin receptor, with a lower affinity.

On the other hand, majority of IGF-I actions are mediated through the union of IGF-I to its putative receptor, IGF-IR, a tyrosine kinase that is one of the most potent natural activators of Akt pathway, involving among others: mTOR, MAPK, GSK3β, FOXO, HDM2, Grb2 and Shc systems, all of them closely related with cell survival, growth and proliferation [15-17]. However, IGF-I can also bind to the insulin receptor (with a lower affinity), as a secondary via through which this hormone mediates some of its metabolic functions [6], due to their high homology. Complementarily, insulin can also bind to IGF-IR with a lower specificity than insulin receptor (Figure 1).

Under this scenario, a review about the increasing list of IGF-I roles, both in physiological and pathological conditions, and its therapeutical potential, arises as a promising field of work.

## Physiological roles of IGF-I

IGF-I is a relevant hormone both in embryological and postnatal states. Although it is mainly produced by the liver, virtually every tissue is able to secrete IGF-I for autocrine/paracrine purposes [18].

Pituitary (GH) and liver (IGF-I) establish negative feedback mechanisms common to any other endocrine gland. The pituitary somatotrophs (GH-secreting cells) are under a delicate controlled balance between stimulatory growth horomone-releasing hormone and inhibitory somatostatin, both generated by the hypothalamus as a result of systemic and cortical neurogenic, metabolic, and hormonal factors [19]. In another hand, IGF-I inhibits GH secretion acting on the hypothalamus by two feedback mechanisms: firstly, inhibiting GH gene expression [20] and secondly by stimulating the secretion of somatostatin [21,22], that inhibits GH production.

Secreted GH can exist in both free and bound states by the GHBP (the secondary domain of the GH receptor) [19]. Also, activation of liver GH receptor, promotes IGF-I synthesis which, in turn, is released to the circulation and can be found in its free form but mainly bound to IGFBPs (overall IGFBP-3, which binds ~90% of circulating IGF-I) [23].

The role of IGF-I in physiological conditions is still being uncovered and continuously unfastened from GH actions as an independent, self-sufficient peptide. For example, it is known that GH and nutrition are the major factors that regulate hepatic IGF-I expression, as well as in other organs [24,25]. However, in some other tissues, IGF-I expression appears to be regulated by tissue specific trophic factors, as for example in uterus, where estrogens (and not GH) stimulate IGF-I expression [26], while follicle stimulating hormone is a major IGF-I regulator in ovary [27].

**Figure 2:**
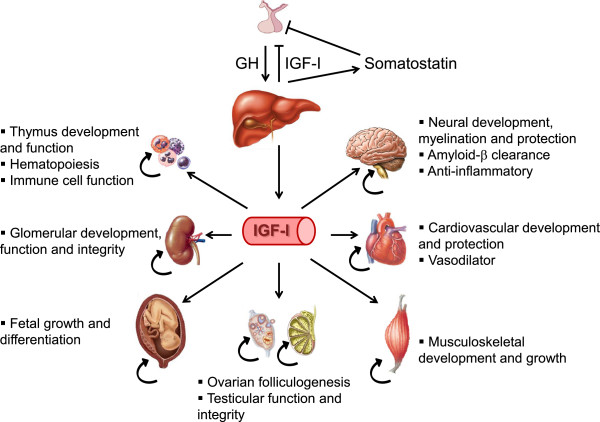
**GH/IGF-I axis and targets.** Pituitary GH interacts with GH receptors in hepatocytes increasing IGF-I secretion for endocrinological purposes in different organs, although an autocrine/paracrine IGF-I production by those organs is also present.

In an attempt to provide a coherent and integrated review of certain physiological conditions where the role of IGF-I has been well established, we have summarized them in this review (cfr. Figure 2).

### Body growth

IGFs have been shown to play a very important role in fetal growth and differentiation [24,28,29], although their pattern of expression and abundance varies among organs. For instance, in fetal liver, kidney and heart are lower than IGF-II, while they progressively increase after birth (as serum IGF-I concentration does). However, expression of IGF-I in fetal lung, muscle, and stomach is higher than it is postnatally [29].

Of interest, while the actions of IGF-I after birth are being continuously recognized, the physiologic role of IGF-II is still poorly understood at this stage [1,30]. Interestingly, it was reported that GH is not required for normal intrauterine growth, a finding supported by evidence that GH deficiency/insensitivity does not associate with a significant reduced size at birth [31-35]. In contrast, inactivating mutations of IGF-I or its receptor, have clearly established that IGF-I is a major regulator of intrauterine growth [36-41]. Thus, these findings suggest that the stimulatory role of IGF-I on intrauterine growth is GH-independent.

With this perspective, Daughaday et al. [5,42] proposed the somatomedin hypothesis for the postnatal growth, where GH stimulated skeletal growth by stimulating liver production of IGF-I, which in turn promotes longitudinal bone growth in an endocrine manner. However, a noteworthy direct action of GH on bone growth has also been reported [43,44]. Nevertheless, the discovery of an extra-hepatic IGF-I production in the following years [18,45], made it necessary to spand the conception of the GH/IGF-I axis, being well accepted nowadays that pituitary GH is able to induce IGF-I synthesis in both liver (~75%) and other tissues [23,46,47], which subsequently, is able to act in an endocrine/paracrine/autocrine manner.

So, taking into account all these data, we can conclude that both GH and IGF-I has independent and synergic effects in promoting postnatal body growth. This idea was also confirmed by comparing the weight of transgenic mice with GH receptor inactivation, IGF-I knockout and the double inactivation [36]. As expected, double knockout mice presented a more severe reduction in body length than the other two models (~20% more).

Finally, GH and IGF-I (both liver/endocrine and locally produced) are essential for normal body growth. The role of other molecules for this control is also important and may increase the complexity in the understanding of these mechanisms. Acid-labile subunit (ALS) and IGFBP-3 are two proteins that bind IGF-I (~90% of total serum IGF-I) in a ternary complex which transports and prolong IGF-I half-life in circulation [23]. Although the liver is *also* the principal source of circulating IGFBP-3 and ALS, other tissues have been proposed to produce these factors [48,49]. The importance of IGFBP-3 relies also on its capability to act independently from IGF-I, regulating growth, apoptosis and metabolism of target cells [50-52]. Therefore, the expression of ALS and IGFBP-3 in non-hepatic tissues and possible IGF-I-independent effects of IGFBP-3, should be considered when analyzing findings. To go through IGFBP-3 roles/effects in depth the review by Ohlsson C et al. is recommended [23].

### Central nervous system (CNS) development

Although the action mechanisms of IGF-I in neurons have not been fully elucidated, it has been shown that IGF-I stimulates the autophosphorylation of IGF-IR [53] in a different manner to insulin [54]. Furthermore, as it will be further discussed, we have reported a neuroprotective role for IGFs associated to mitochondrial protection and antioxidant defenses in aging animals [55-58]. Both mechanistic pathways, that may be linked, are currently being studied in more detail.

IGF-I is harmonically produced by peaks that coincide with periods of neuron progenitor proliferation and differentiation, neuritic outgrowth (increasing the number of dendrites, axonal cones, synapse number…) or post-injury conditions [59]. However, the possibility of IGF-I to influence neural stem cells (NSC) is still on debate despite the fact that IGF-I and IGF-IR are expressed in cultured NSC [60,61], and that in response to IGF-I, cultured NSC proceed toward specific lineages, such as neurons [62] or oligodendrocytes [63].

Interestingly, not only neural-produced IGF-I is involved in these processes. It was first reported 20 years ago that systemic IGFs could cross the blood–brain barrier (BBB), when labeled IGFs were infused into the common carotid arteries of adult rats, and they were later on detected in the choroid plexus, median eminence, brain arterioles, and parenchyma [64]. In fact, this work was based on previous data confirming the presence of IGF-I receptors in brain capillary endothelial cells (which constitute the BBB), and their role in internalizing IGFs to the CNS from the circulation [65,66]. A more recent work by Torres-Aleman’s group, elegantly demonstrated that this process is initiated by the release of glutamate at active regions, triggering two secondary processes: a vasodilation to increase local serum IGF-I availability and an increased activity of matrix metalloprotease 9, together with cleavage from IGFBP-3. The combined action of these events results in an increased local availability of free serum IGF-I, which is then transported by transcytosis using an endothelial transporter (lipoprotein related receptor 1) dependent mechanism [67]. This research may shed light on previous data identifying liver-derived IGF-I as one of the main factors in regulating the clearance of brain amyloid-β (Aβ) levels [68] and its potential implications in Alzheimer’s Disease (what will be further discussed in III.4.3.). However, it is noteworthy that there is no significant correlation between serum and cerebrospinal fluid IGFs concentrations [69] and therefore, it seems that systemic IGFs are not a major source of IGFs for CNS.

On the other hand, little is known about those factors and mechanisms that regulate IGF-I expression in the brain, but there is evidence, however, that growth factors (i.e. GH, epidermal growth factor, basic fibroblast growth factor) [70-72], nutrition [73,74], and injury (hypoxic/ischemic, stereotactic, electrolytic and cryogenic injuries, as well as induced demyelination and experimental autoimmune encephalomyelitis) [75-81], influence its *in vivo* brain expression. Nevertheless, IGF-I mRNA abundance is reduced in the brain of hypophysectomized rats and, intracerebral infusion of GH restores IGF-I mRNA to 80% of normal levels [70], which points out that GH has a clear role in modulating brain IGF-I.

Additionaly, IGF-I may promote proliferation and/or survival of oligodendrocytes and their precursors, but it also could be involved in the modulation of BBB permeability [81]. The latter would limit accessibility of T lymphocytes and soluble destructive immune factors to the brain. Other possibilities are also plausible, as for instance, IGF-I may influence the peripheral immune reaction, what in turn could reduce CNS inflammation, demyelination, and BBB permeability [82]. These findings, taken together with the neuroprotective actions of IGF-I [55,56], suggest that astrocytes are relevant in ameliorating brain injury.

In another hand, *in vivo* experiments with transgenic mice have clarified some aspects about the topic. Transgenic (Tg) mice that overexpress IGF-I in the brain exhibit postnatal brain overgrowth without anatomic abnormality (up to 85%) via an increase in cell number [83] and myelination [84]. A complementary experiment [83] excluded the possibility of GH in directly promoting these effects, since GH-overexpressing Tg mice did not exhibit those changes. However, as stated before, a role for GH in brain growth cannot be underestimated, since GH-deficient mice have significantly smaller brains than normal mice [23]. Consistently, transgenic mice with ablated IGF-I expression barely survive postnatally. Survivals have very small brains (−60% of normal size) but remain morphologically normal [85]. These brains are characterized by a paucity of white matter owing to markedly decreased myelination [86] and an apparent decrease in the number of axons [85].

These IGF-I actions, taken together with its neuroprotective effects following CNS and peripheral nerve injury, suggest that it may be of therapeutic benefit in a wide variety of disorders affecting the nervous system.

### Liver regeneration

The liver is the main source of circulating insulin-like growth factor I, accounting for ~75% of circulating IGF-I levels secondary to the GH stimulation on hepatocytes [23,46,47]. Of interest, although liver-derived IGF-I has endocrine effects on extrahepatic tissues, there are only few data regarding local effects of this hormone in the liver [87] probably due to the very low amount of IGF-I receptors on the hepatocytes membrane [46,88,89]. However, there are IGF-I receptors on the nonparenchymal cells [89,90] and it has been reported that IGF-I stimulates both DNA synthesis [91,92] and the production of hepatocyte growth factor (HGF) in hepatic stellate cells *in vitro*[87].

The lack of IGF-I receptors on hepatocytes would also mean that liver-derived IGF-I would be unable to stimulate liver growth during adulthood. Accordingly, mice with liver-specific IGF-I deficiency, instead of displaying decreased hepatic growth, showed disproportionally large livers, likely due to direct stimulation by an unsuppressed GH secretion [47,93]. In line with this finding, GH receptor deficient mice have reduced relative liver weight [94], and transgenic mice overexpressing GH presented disproportional growth of the liver, whereas this is less apparent in mice overexpressing IGF-I [95,96].

Nevertheless, during liver regeneration, where it is required an explosive burst of hepatocyte renewal (i.e. after partial hepatectomy), IGF-I may play a role in supporting hepatocyte proliferation and accelerating DNA synthesis [97,98], together with IL-6, TNF-α, HGF and TGF-α/EGF [99,100]. It remains unknown why IGF-I stimulates liver regeneration more effectively than growth of intact liver.

### Gametogenesis

#### Ovarian folliculogenesis

The process of folliculogenesis can be divided into three developmental phases: I) preantral follicle growth: primordial to primary follicle transition, and formation and growth of secondary follicles; II) basal antral follicle growth: antrum formation and development of early antral follicles to the gonadotrophin-dependent stage; and III) terminal antral follicle growth: development of antral to preovulatory follicles [101].

The involvement of the IGF system as intraovarian regulators of folliculogenesis has been intensively studied in a variety of mammal species, and it is now established that the ovary is a site of IGF-I gene expression and reception [102]. However, this huge amount of data could be somehow distractive since different species may produce distinct IGFs (or their relative binding proteins and receptors) at different stages of follicular development. For this reason, in this review, we will focus on murine and primate data.

In primate, mRNA expression patterns of IGF-I and its putative receptor have been deeply studied during folliculogenesis. IGF-I is expressed in primordial follicles, primary follicles, secondary follicles and growing antral follicles (oocyte and theca), but not in preovulatory follicles (mural granulose and theca) [101]. Of interest, IGF-IR mRNA is temporally consistent with the IGF-I expression, except from the mural granulose cells, where IGF-IR production is preserved, suggesting a paracrine/endocrine dependence for the IGF-I effects at this level [101].

The lack of information regarding the role of IGFs in a specific time point of human folliculogenesis, hinder its correlation with the expression patterns of IGF-I. Fortunately, murine models provide us a useful tool to elucidate its possible implications. This approach suggest that IGF-I may play a role at different stages of follicular development: a) initiation of growth of the primordial follicle; b) at a secondary follicle stage, IGF-I may be involved on induction of FSH-R expression on granulose cell and their differentiation, theca cell survival and cortical granules formation in oocytes [103-106]; and c) at antral follicular stage, IGF-I may increase follicle sensitivity to gonadotrophin, oocyte maturation and LH-R expression in granulose and theca cells enhancing their proliferation and steroidogenic activity [107-111]. In humans, IGF-I also stimulates vascular endothelial growth factor production by granulose cells [112].

Despite recent progresses, the precise mechanisms underlying ovarian follicular growth are not yet fully elucidated. In most mammalian species studied, although GH and IGFs do not appear to be required for primordial to primary follicles transition, they are responsible for promoting secondary follicle growth and antrum formation. In brief, GH enhances the development of small antral follicles to the gonadotrophin-dependent stages and stimulates oocyte maturation, whereas IGFs increase granulose cell proliferation, steroidogenesis and oocyte growth in most mammalian species [101].

#### Testicular function

Although it is well established that testicular function is mainly controlled by the gonadotropins LH and FSH [113,114], there is now considerable evidence pointing to locally produced factors as important key regulators of testicular function [115]. Among those, IGF-I has been reported to be a potent candidate due to its para/autocrine functions. Immunostainable IGF-I has been found in adult human testes [116]. Cultures of Sertoli and Leydig cells from adult rats and immature pigs secrete immunoreactive IGF-I into the medium, and this secretion is enhanced by FSH (Sertoli cells) or LH (Leydig cells) [117,118]. IGF-IR has also been found on human, pig, and rat Leydig cells [119-121], where it enhances the differentiated functions of Leydig cells [122,123].

The crucial role of IGF-I in the development and function of Leydig cells was obtained from studies in IGF-I knockout mice [124]. The testes of these animals were reduced in size and although epididymides were overall nearly allometric to the reduced body weight, the distal regions of the duct, vas deferens, seminal vesicles, and prostate were vestigial. These transgenic mice showed significantly reduced plasma testosterone levels (18% of normal) [124] and the IGF-I deficiency was correlated with an ultrastructural analysis of mutant Leydig cells revealing a significant developmental delay, with fewer and smaller Leydig cells than normal. Importantly, it is noteworthy that those reduced testosterone levels in serum were inadequate for perinatal androgenization. And secondly, androgen deficiency in the mutants can be correlated with an apparently retarded differentiation of Leydig cells (in concrete, their second phase) [124]. *In vitro* studies also suggested a relationship between IGF-I and LH-androgen production in rodents [125], through a direct effect on Leydig cells.

Consistently, a large number of studies have previously suggested that the IGF system is involved in mammalian reproductive functions [108,126,127]. First, it has been emphasized the importance of insulin receptor family for the induction of testicular differentiation by Sry-dependent processes [128]. In addition, IGF-I regulates the expression of key steroidogenic enzymes during prenatal development, which in turn lead to establishment of the male phenotype and fertility [129]. Moreover, male gonads cultured in the presence of IGF-I increased testosterone production during testicular development [130]. Postnatally, *in vitro* studies have also demonstrated that the IGF-I regulates the expression rate of genes encoding steroidogenic enzymes that involved in the biotransformation of steroid hormones in the testis [131-133]. Secondly, a role for IGF-I on sperm number has been reported since in IGF-I deficient mice there is a dramatic reduction of sperm number [124]. Moreover, the vast majority of these males that were caged with wild type females did not exhibit mating behavior.

In another hand, it is notable that the role of GH, if any, on the regulation of intratesticular IGF-I may not be significant, since GH receptor gene may not be expressed in the testis [134,135] and GHI animals are fertile (normal steroidogenesis and spermatogenesis) [136,137]. Interestingly, despite GH deficiency and low serum IGF-I concentration, they exhibit normal levels of testicular IGF-I [119]. Thus, in addition to being apparently GH-independent, the testicular functions of IGF-I seem to be served by its local production (autocrine/paracrine action) without a major endocrine contribution by the circulating form of this factor.

### Kidney development and function

Several lines of evidence support the role of the GH/IGF-I system in normal kidney development and function. IGFs, IGFBPs and IGF receptors (along with GH receptors) are all expressed in specific locations along the nephron, suggesting that IGFs have paracrine and autocrine actions at these sites [138,139].

Both IGF-I and the IGF-IR are expressed in glomerulus development being their patterns of expression disrupted in animal models and in human examples of renal disease [140]. Indeed, it has been demonstrated a role for IGF signaling in maintaining glomerular integrity, by preserving podocytes and the glomerular basement membrane from damage. Consistently, IGF-I administration to rodents increases kidney growth, renal blood flow and glomerular filtration rate (GFR) [138,141,142], and similarly, GH and IGF-I also increase renal blood flow and GFR in humans [143], suggesting that IGF-I may be a physiologic regulator of renal function.

Furthermore, a possible role for the IGF system in compensatory renal growth was proposed since renal IGF-I levels are increased in the remaining kidney following uninephrectomy and compensatory renal growth [144] in an age-dependent manner [145]. However, by using IGF-I deficient mice, it has been recently reported that uninephrectomy in these mice induces a significant and proportional increase in renal mass, as compared to normal mice, despite markedly decreased kidney IGF-I levels and no significant changes in receptor phosphorylation [146]. Therefore, the implications of IGF-I in this process may be elucidated in following years.

### Cardiovascular development

Cardiovascular system is an important target organ for GH and IGF-I actions. There is evidence that IGF-I and its receptor are expressed in the myocardium and both aortic smooth muscle and endothelial cells [45,147-149], being all of them more sensitive to IGF-I than to insulin [150,151]. In addition, cardiac IGF-I production increases in response to GH [45]. Consequently, there are different possibilities of direct actions of GH as well as endocrine or autocrine/paracrine effects of IGF-I on the cardiovascular system.

Previous studies indicate that IGF-I is a potent vasodilator [152], and that this effect may be partly mediated by increased NO release from the endothelium [153,154]. Accumulating evidence would also suggest that insufficient IGF-I levels play a role in vascular diseases such as atherosclerosis and restenosis [155], what will be discussed in further sections (cardiovascular diseases).

### Immune modulation

The potential relationship between immune function and growth factors such as IGF-I has remained poorly characterized until recently. However, the realization that diverse regulatory pathways often converge, motivated a number of studies that eventually demonstrated the importance of GH, IGF-I, and IGF-IR in many processes of immune function [156].

Complex interactions between cytokines and growth factors, including IGF-I, has been properly reviewed by O’Connor et al. [157]. In brief, pro-inflammatory cytokines seem to damp several components of the IGF-I pathway. Many of the cytokines share common signaling components, such as Erk1/2 MAPK.

The role of IGF-I on thymus development and function, hematopoiesis and immune system reconstitution is well documented [158-161].

The role of IGF-I on different immune cell lineages has also been reported. Both IGF-I plays important roles in T lymphocytes development and function. Specifically, IGF-I can increase the number of CD4^+^CD8^+^ immature T cells in rat thymus and spleen [162], promotes T cell survival [163], proliferation, chemotaxis and maturation, and blocks spontaneous and induced programmed cell death [163,164], although it has also been reported to block IL-2-dependent lymphocyte growth and function [165]. The possibility for IGF-I to determine how T-cell compartments are filled throughout life remains an open question. However, given the importance of IL-7 in that process and how IGF-I potentiates the actions of IL-7 in pro-B cell expansion [166], a similar influence on T cells seems likely.

Aging rodents exhibit diminished responsiveness to pathogens. This shift is associated with reduced cellularity and significant thymus involution [167,168]. As will be further discussed in section “Aging and age retaled diseases”, IGF-I and GH levels also decrease with aging (somatopause) [169], reason why a potential strategy for reversing these senile changes in thymic vitality may involve administration of either GH or IGF-I, which have been examined for their potential to expand T-cell populations in animals [170].

B cells play diverse roles in immune function by virtue of their further differentiation into immunoglobulin-secreting plasma cells, generation of cytokines, and their importance in antigen presentation. IGF-I has been reported to drive B-cell differentiation, to enhance IL-7-dependent B-cell proliferation in parallel with c-kit ligand [171], as well as to potentiate IL-7 promotion of pro-B-cell expansion [166]. When administered *in vivo*, IGF-I also enhances the population of intrasplenic B cells through increased proliferation of mature cells [172,173] together with an influence on antibody expression and class switching by plasma cells [174].

Human macrophages and granulocytes are also sensitive to IGF-I by displaying IGF-IR [156]. It was documented that IGF-I attenuated spontaneous apoptosis in these populations [175].

Finally, neutrophils seems to be a potential target for IGF-I actions, since it was able to delay Fas-mediated apoptosis through the PI3K pathway. Moreover, this effect was conserved even in the presence of pro-apototic cytokines, suggesting that it may play a dominant role, even within the context of an active inflammation [156].

## Conditions of IGF-I deficiency

An increasing list of animal models has been reported highlighting the role of this molecule in many different organs and systems. In this review, we will focus on the best characterized models of IGF-I deficiency, where the substitutive therapy may be an effective strategy.

### Intrauterine growth restriction (IUGR)

Fetal growth is a complex process involving maternal, placental, and fetal factors from genetic, environmental, and nutritional nature. Intrauterine growth restriction is an important obstetric issue affecting ~5% of pregnancies and refers to a fetus that has not reached its growth potential [176]. The growth-restricted fetus/newborn is characterized by an increased fetal and neonatal mortality and morbidity [177,178] and an increased risk of clinical disorders in adult life, such as cardiovascular disease, diabetes and obesity [179,180].

In the prenatal period, differences between GH and IGF-I are clearly shown. Whereas GH insensitivity, both in humans and in transgenic mice, have only mild retardation of growth at birth as previously stated [31-35], IGF-I deficiency in gestational state reveals serious postnatal growth retardation, as has been reported both in humans and in transgenic animal models of IGF-I deletion [36-40]. Interestingly, in contrast to growth hormone insensitivity (GHI), the IGF-I deficient animals are neurologically impaired, as was also reported in a single patient with a defect in the IGF-I gene [40]. Thus it appears that IGF-I is necessary for normal brain development in uterus while GH insensitivity may be recovered by an intrauterine GH-independent production of IGF-I [181,182].

Fetal progress is widely controlled by the oxygenated blood reaching the uterine circulation, the placental integrity and function, and the fetus ability to get the required nutrients [183]. It has been estimated that progenitor’s genes account for only 20% of the variation of human birth weight. Nevertheless, majority of the variation (62%) is due to the intrauterine environment [184]. Placentas from IUGR pregnancies have been shown to have poor invasion of the trophoblastic cells into the maternal decidual tissues, particularly the maternal spiral arteries [185,186]. Studies into the pathological process of IUGR have pointed to an abnormal placental function as a common mechanism [187]. However, it is known that the placental dysfunction is often gradual and it can occur much earlier than any demonstrable IUGR [188], thus difficulting the resolution of this hypothesis.

In the same way, IGFs control growth directly, and circulating IGF-I appears to be virtually independent of fetal GH secretion [182]. However, under this condition, placental growth hormone may take this role as the prime regulator of maternal serum IGF-I during pregnancy [189], being of particular interest the positive expression of IGF-IR in placenta [190] and the lower expression of placental-derived IGF-I during IUGR [191]. In general, endocrine milieu of the human fetus with growth retardation is also characterized by low circulating levels of insulin, IGF-I, IGF-II, and IGFBP-3, and high levels of GH and IGFBP-1 [185,192,193]. At this point, an elegant study in zebrafish demonstrated that knockdown of IGFBP-1 significantly alleviated the hypoxia-induced growth retardation and developmental delay. And consistently, overexpression of IGFBP-1 caused growth and developmental retardation under normoxia conditions [194].

In the last years, it is being proposed a role of fetal programming for an altered GH/IGF axis in IUGR, constituting the so-call Thrifty Phenotype Hypothesis [185], with an already proven inverse association between IGF-I levels at 9 months and 17 years. Under this perspective, GH/IGF-I axis may be programmed early in life. This fetal programming could be involved in, at least, two pathological conditions in later life, as insulin resistance and hypertension. Firstly, children with IUGR show an impaired GH/IGF-I axis, which might be contributing to reduced insulin sensitivity and IGF-I resistance, as higher basal and GH-induced IGF-I levels are required to achieve a growth velocity similar to that of other children, what secondarily leads to a compensatory hyperinsulinemia to counteract insulin antagonistic effects of GH [195] and, an impaired regulation of glucose transporter-4 expression by insulin in muscle and adipose tissue [196].

In another hand, fetal responses to IUGR-related hypoxia include downregulation of insulin, IGF-I, and IGF-II and increased expression of inhibitory IGFBPs. Hypoxia also activates the hypothalamo-pituitary-adrenal (HPA) axis, raising plasma levels of adrenocorticotropic hormone and cortisol, another mechanism that regulates IGFBP expression [197]. Moreover, as previously stated, kidney growth is under IGF-I control; and a reduced IGF action, parallel to increased cortisol levels, results in a smaller number of glomeruli [198]. Alterations in the renin-angiotensin system are also frequent, probably downstream to activation of the HPA axis. These changes together with compensatory responses for the reduced kidney function probably account for the predisposition to adult hypertension.

### Laron syndrome (LS)

In 1966, Zvi Laron et al. described the first condition of IGF-I deficiency as a new type of dwarfism indistinguishable from genetic isolated GH deficiency, but with unexpected high serum GH levels [199,200] and inability to synthesize IGF-I and other related molecules, as IGFBP-3 [34,35,201]. This heterogeneous condition was finally named as Laron Syndrome or primary Growth Hormone insensitivity (GHI), and it includes: GH receptor deficiency (the most common), GH-GH receptor signal transduction defect, IGF-I synthetic defect, IGF-I receptor deficiency and IGF-I/IGF-I receptor signal transduction defects.

Epidemiologically, this pathological entity is closely related to an ethnic origin (>90% of cases) [19]. Clinically, overall growth in uterus is slightly shorter at birth in LS (42–47 cm) than in healthy babies (49–52 cm), suggesting a potential role of IGF-I in controlling intrauterine linear growth [34] as will be discussed ahead. This condition is more dramatic throughout childhood, where both skeletal maturation and organ growth are retarded [19,34,202] probably due to a lower impact of GH on gestational growth as compared to IGF-I [31-40]. These growth abnormalities in LS patients without IGF-I substitutive treatment includes postnatal average growth rates of one-half the expected during the first years of life [19], a small brain (with prominent forehead, reduced vertical dimension of the face and hypoplasia of the midfacies and the nasal bridge), a small heart and acromicria [203] together with underdevelopment of the muscular system that delays walking in three-fourth of patients [204,205], osteopenia at all stages (despite normal sex hormone status) with increased occurrence of avascular necrosis of the femoral head [206], impair and weaken skin, hair and nail growth [207], blue sclera due to the decreased thickness of its connective tissue, allowing visualization of the underlying choroid [205], a puberty delay from 3 to 7 years [206], retardation in the maturation of dentition [206] and high-pitched voice [208]. Of interest, normal reproductive function and behavior are widely preserved [202].

This condition of IGF-I deficiency highlight the critical role of IGF-I on brain development and function, as also will be stated in section “Neurodegenerative diseases”.

Animal models of GHI are available since 1997 [31], helping us to better understanding the patho-physiological changes and potentially improved strategies for treating these patients. Nowadays, this is the unique condition of IGF-I deficiency where rhIGF-I is approved (as will be stated in “Current therapeutic options” section).

### Chronic liver disease

Cirrhosis is a consequence of chronic and diffuse liver disease characterized by replacement of liver tissue by fibrosis, necrosis and regenerative nodules, leading to loss of functional liver mass. Cirrhosis is most usually caused by alcoholism, hepatitis B and C, and fatty liver disease, among other possible causes [209].

Most common complications of advanced cirrhosis include jaundice and coagulopathy from hepatocellular insufficiency, gastrointestinal bleeding from esophageal varices, ascites, hepatorenal syndrome, spontaneous bacterial peritonitis, liver-related encephalopathy, and malnutrition [209]. Although survival in patients with compensated cirrhosis is relatively high (90% at 5 years after diagnosis), occurrence of complications worsen this scenario to 30% at 3 years [210,211].

Liver cirrhosis was firstly associated with IGF-I in the late ‘80s, proposing this hormone as a good indicator for functional hepatocellular capability [212-214] with a marked decline from early cirrhosis stages (Child-Pugh A) [215]. Since then, the idea of liver cirrhosis as a condition of IGF-I deficiency during adult age has been yearly consolidated from a number of publications establishing the origin of this lack from a decrease in GH receptors seen in cirrhotic livers [216] and a progressive reduction of liver synthesis capability from decreased hepatocellular mass in advanced stages [209]. Furthermore, a marked decline of IGF-I has also been related to a higher probability of hepatocarcinoma [217] and poorer prognosis in patients requiring liver surgery [218]. As a result IGF-I levels are considered of prognostic value regarding survival in cirrhotic patients [215,218].

The availability of animal models for experimental liver cirrhosis (carbon tetrachloride, thioacetamide, bile duct ligation, D-galactosamine…) helped us to better elucidate the role of IGF-I in this pathology. In concrete terms, our group of work has reported that cirrhotic animals treated with rhIGF-I showed: increased food ingestion and efficiency with increased nitrogen uptake and balance (resulting in an increase in muscle weight) [219], normalization of intestinal amino acid and sugar absorption [220,221] (including in animals with advanced cirrhosis and ascites [222]), glucose metabolism [223], reduced portal pressure, endotoxemia and bacterial translocation [224], improved osteopenia both in compensated and ascetic cirrhosis [225,226] and testicular morphology and function [227,228], recovered somatostatinergic tone [229] with improved liver function (increased albumin and coagulation factor levels) and decreased hepatic fibrosis [223]. We have also reported that the hepatic restoration was associated to antioxidant, antiapoptotic, antifibrogenic and mitochondrial-protective effects of IGF-I [230-234].

### Aging and age-related diseases

Aging is a universal, intrinsic, irreversible, heterogeneous and multidimensional process of progressive involution characterized by a gradual loss of physiological functions that increases the probability of death. Although related, longevity is different from aging, since the first one is simply considered as the length of the life span independent of the biological aging process. Average lifespan has being (hopefully) continuously growing: from ~27 years in the Greco-Roman era, to ~47 years in 1900, and ~77 years by the end of 20^th^ century [235]. However, it is very interesting that maximum lifespan (longevity) has not dramatically changed and seems to rest at about 120 years, despite the increase in the number of centenarians [236].

Circulating GH and IGF-I levels are maximal during peripubertal growth and early adulthood; however, they progressively decline with age [169]. This decline during human aging is sometimes referred to as somatopause, in analogy with the menopause and andropause. Reduced GH/IGF-I secretion in the elderly is believed to be responsible for or contribute to many symptoms of aging, including loss of muscle mass, increased adiposity, reduced bone mineral density, and decline in energy levels, along with alterations in psychological indicators of the quality of life [237].

**Figure 3:**
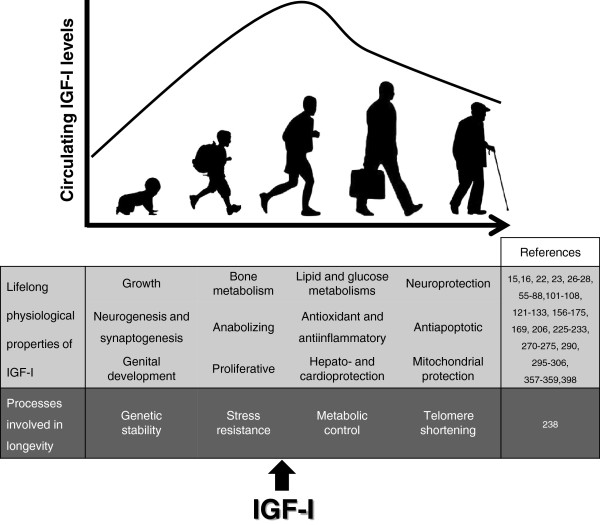
**Lifelong beneficial properties of IGF-I.** Evolution of IGF-I circulating levels and its pluripotent roles along different stages of human development and aging.

There are diverse theories of aging [238] that basically point to few broad physiological processes important for longevity: genetic stability, telomere shortening, stress resistance and metabolic control. Interestingly, IGF-I is somehow related to all of them (Figure 3). Firstly, assuming that mitochondria are the main source of endogenous free radicals [239,240], it has been previously reported that species with higher metabolic rates have shorter maximum lifespan due to superoxide anion radical accumulation that lead to cell damage, hastening aging [241]. At this point, we have previously shown that IGF-I is a main character in restoring mitochondrial dysfunction during aging by increasing mitochondrial membrane potential, reducing oxygen consumption, and increasing ATP synthesis what in turn minimize the c-cytochrome release to the cytoplasm and subsequently promote neural survival by decreasing caspase-induced apoptosis [55,56], in agreement with *in vitro* reported works from others [242-244]. Furthermore IGF-I’s antioxidant capability in brain cortex and hippocampus was assessed as improving antioxidant enzymes activities (superoxide dismutase, catalase and glutathione peroxidase) and parameters of oxidative damage (MDA and PCC) [55,56]. Thus, by improving mitochondrial function and decreasing oxidative insults, IGF-I may be protecting DNA, proteins and lipids.

Secondly, IGF-I has been proposed as an index of healthy aging, due to the finding that it directly correlates with the leukocyte telomere length [245,246], a biomarker of human aging associated with increased risk of developing vascular diseases, metabolic disorders, and other age associated phenotypes [247,248].

**Figure 4:**
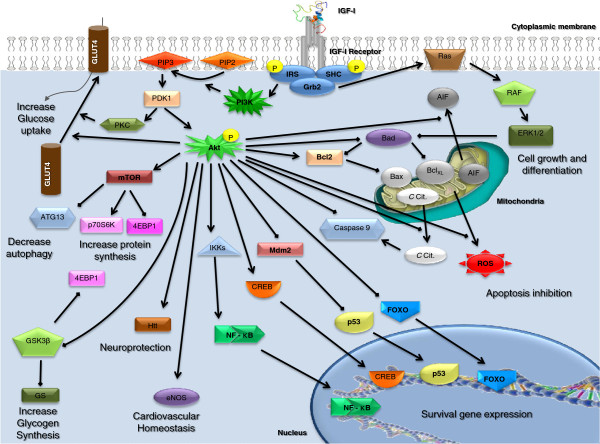
**IGF-I receptor pathway.** Activation of IGF-IR leads to a cascade of related molecules involved in previously described actions of IGF-I (survival, cell growth and differentiation, cytoprotection, glucose metabolism, etc).

And thirdly, another aspect where IGF-I may play a role in delaying aging symptoms is by controlling metabolism, together with insulin [249]. The best characterized intracellular substrates for the insulin and IGF-I receptors are the insulin receptor substrate proteins 1 to 4 [250]. Following tyrosine phosphorylation, each of these substrates associates with Src homology 2 (SH2) domains of intracellular molecules to generate downstream signals (cfr. Figure 4). The two most important SH2 molecules at this point are the adaptor molecule Grb2 and the enzyme PI3K [251,252]. Grb2 links insulin action to the Ras-MAPK pathway, stimulating cell growth and differentiation. PI3K, on the other hand, is responsible for the insulin-like actions of the hormones by activating Akt/protein-kinase B (PKB) and protein-kinase C (PKC), which subsequently leads to activation of p70 S6K and glycogen-synthase kinase 3 [252]. This, eventually results in stimulation of glycogen, lipid and protein synthesis, as well as in glucose transporter translocation to the plasma membrane with an increase in glucose transport [253]. Importantly, Akt/PKB also phosphorylates forkhead transcription factors of the FOXO subfamily, and this leads to their inactivation and retention in cytoplasm [254,255] resulting in a reduced transcriptional activity [256]. As a final step, depending on the nature of the activation signal, FOXO can regulate apoptosis [257], cell cycle [258], differentiation [256], or the expression of genes involved in DNA repair [259] and oxidative stress resistance [260].

In addition, insulin sensitivity normally decreases during aging, and insulin resistance is a well established human risk factor for a variety of illnesses that affect morbidity and mortality among the elderly, including hypertension, atherosclerosis, obesity, diabetes and neurodegenerative disorders [261-265]. In our group, we have demonstrated that low doses of IGF-I restored circulating IGF-I levels, which improves insulin resistance and lipid metabolism in aging rats [55], thus becoming a potential beneficial agent to prevent insulin resistance-related pathologies.

As stated before, aging and longevity are different concepts. And this is also evident at the IGF-I function level. Despite all the beneficial effects of IGF-I described up to now and the following ones reporting its role in aging-related pathologies as musculoskeletal, neurodegenerative, cardiovascular diseases, IGF-I has been paradoxically negatively related to lifespan [266,267], probably due, at least in part, to its downstream molecule Akt and its interactions with FOXO, mTOR and SIRT-1, among others [268,269]. These outcomes demand much more work to elucidate the apparently contradictory actions of IGF-I on aging and longevity.

#### Cardiovascular diseases (CVD)

Cardiovascular diseases remain the biggest cause of deaths worldwide, though over the last two decades, cardiovascular mortality rates have declined in many high-income countries. At the same time cardiovascular deaths and disease evolution have increased at an astonishingly fast rate in low- and middle-income countries due to the increase in obesity, sedentary lifestyles and unhealthy habits [262].

Over the last years, low circulating IGF-I levels have been correlated with an increased risk for CVD in humans [270-274]. In cross-sectional studies, low circulating IGF-I levels were found to be associated with angiographically documented coronary artery disease [275] and may predict fatal ischemic heart disease [272], a significantly increased risk of ischemic stroke and congestive heart failure in elderly patients [276,277], as well as a worse prognosis of recovery after an acute myocardial infarction [278]. Additionally, it has been reported a positive correlation between circulating IGF-I levels and both coronary flow reserve [279] and successful cardiovascular aging in healthy centenarians [280].

Available data from transgenic mice with liver-derived IGF-I deficiency clarified that IGF-I deficiency *per se* can promote the development of an accelerated cardiovascular aging phenotype. Particularlly, contractility of cardiac myocytes is significantly impaired [281,282] together with a deficient compensatory hypertrophic response, following experimental aortic constriction [281]. This animal model also exhibit a dysregulation of Nrf2-dependent antioxidant responses in the vasculature, which promotes the development of marked endothelial dysfunction and endothelial apoptosis in the presence of increased oxidative stress [281], mimicking the aging phenotype.

Consistently, a complementary strategy showed that in aged mice, where IGF-I levels are decreased, cardiac overexpression of IGF-I significantly improved cardiomyocyte contractile function [283], attenuating oxidative stress-mediated protein damage, normalizing Ca^2+^ homeostasis, reversing age-related alterations in the expression of pro-apoptotic proteins, and decreasing the apoptosis rate [284].

In a similar way, a role for locally produced IGF-I have also been documented. It was recently demonstrated that the paracrine IGF-I system confers vasoprotection and cardioprotection [285] and contributes to the maintenance of microvasculature structural and functional integrity. However, vascular paracrine IGF-I system cannot compensate for deficiency of circulating IGF-I [286].

Beneficial effects of the IGF-I/IGF-IR system in cardiac progenitor cells are also starting to be documented. Due to its great potential, this is a new field that deserves more recognition and study. The recent identification of a subpopulation of human cardiac stem cells expressing IGF-IR and secreting autocrine IGF-I with a secondary therapeutic potential for myocardial regeneration [287,288], may be an important step toward this direction. Among others, actions of IGF-I on cardiac stem cells include antioxidant effects, upregulation of telomerase activity, a delay in replicative senescence [288], and migration and homing of cardiac stem cells facilitating neovascularization in damaged hearts [287].

Aging is associated with functional and phenotypic alterations in the microcirculation, including endothelial dysfunction, oxidative stress, chronic low-grade inflammation, and microvascular rarefaction (reduced number and combined length of small vessels in a given volume of tissue) [289]. Our current understanding is that both, circulating IGF-I levels and locally produced IGF-I, contribute to the maintenance of microcirculation functional and structural integrity, increasing NO bioavailability, decreasing reactive oxygen species (ROS) production, and exerting antiinflammatory, antiapoptotic, and pro-angiogenic effects. The mechanisms by which IGF-I reverses and/or prevents microvascular rarefaction and improves tissue blood supply are likely multifaceted, and may involve, among others: 1) Apoptosis, since IGF-I inhibits oxidative stress-induced apoptosis by preserving the functional integrity of the mitochondria [56,290]; 2) Angiogenesis, because IGF-I is known to exert significant pro-angiogenic effects, inducing proliferation of microvascular endothelial cells in culture through HIF-1α and VEGF [291]; 3) Endothelial cell turnover, as it was reported that age-dependent impairment of endothelial progenitor cells is restored by the GH-mediated increase in circulating IGF-I levels [292]; and 4) Oxidative stress and NO bioavailability, since age-related oxidative stress and downregulation of endothelial nitric oxide synthase (eNOS) impair the bioavailability of NO [293], which is likely to contribute to decreased microvascular density. Those facts were confirmed in animal models of IGF-I deficiency as they often exhibit increased ROS production and decreased NO bioavailability, mimicking the vascular aging phenotype [294]. Of interest, treatment of aged rats with IGF-I was shown to upregulate eNOS and improve bioavailability of NO [295], and *in vitro* experiments in cultured endothelial cells demonstrated that IGF-I treatment reduce ROS production and upregulate eNOS [294].

In the same way, it has been postulated that majority of these cardiovascular events related to low IGF-I levels may be due to a possible insulin resistance and accelerated atherosclerosis [296]. Also, the endothelial dysfunction and subintimal modified lipoprotein deposition are frequently consequence of oxidative stress [297] and inflammatory cells activity [298]. Antioxidant and anti-inflammatory effects of IGF-I have been documented to reduce atherosclerotic burden, mainly both via cytokine response modulation (attenuating IL-6 and TNF-α pro-inflammatory responses) [299] and/or eNOS activity regulation [295].

#### Metabolic syndrome

The metabolic syndrome was defined as a combination of clinical features that predispose to enhanced CVD risk, morbidity, and mortality [300]. In some populations, it is present in ~50% of adults [301]. It is thought that an important underlying pathogenetic basis for the metabolic syndrome is insulin resistance and accompanying compensatory hyperinsulinemia [301].

Although without total consensus, six components have been proposed to constitute the metabolic syndrome [300]: Abdominal obesity (or increased waist circumference), atherogenic dyslipidemia (hypertriglyceridemia and low high-density lipoprotein cholesterol concentrations), raised blood pressure, insulin resistance with glucose intolerance, pro-inflammatory state (elevated C-reactive protein) and a pro-thrombotic state (with increased plasminogen activator inhibitor-1 and fibrinogen).

On the other hand, similarities between insulin and IGF-I (molecular homology, shared receptors…) point to the possibility of IGF-I participation in the phenotypic expression of this disorder [301]. The high insulin levels could probably cause a downregulation of IGF-I production by the liver and other tissues, as a compensatory homeostatic mechanism, induced most likely through a differential modulation of IGFBPs production. This might be responsible for the low IGF-I levels seen in association with states of insulin resistance, as the metabolic syndrome [301].

IGF-I’s action on insulin suppression via somatostatine [1] has been tried in diabetes. In type 1 diabetes, where IGF-I and IGFBP-3 levels are decreased [302], substitutive rhIGF-I/IGFBP-3 therapy enhances protein [303] and glucose metabolism by controlling both endogenous glucose output and peripheral glucose uptake [304]. In type 2 diabetic patients, co-treatment with rhIGF-I can significantly reduce glucose levels and insulin requirement [305] while improving glucose tolerance, hyperinsulinemia, and hypertriglyceridemia [306]. Even in nondiabetic subjects, rhIGF-I enhances insulin sensitivity, suppresses lipolysis, clears postprandial lipemia, and increases oxidative and nonoxidative glucose metabolism [223,307,308]. The higher prevalence of insulin resistance and metabolic syndrome in older people compared with younger population may also be attributable, at least in part, to the decline of serum and tissue IGF-I concentrations with advancing age [55,307]. Reduced IGF-I levels are independently associated with glucose intolerance, diabetes, abdominal obesity [309,310] and atherogenic dyslipidemia [311,312]. Overall, these data suggest an important and independent role for IGF-I in protecting against the development of CVD.

Indeed, many of the traditional CVD risk factors, including oxidized low-density lipoprotein [313], hyperlipidemia (hypercholesterolemia or mixed dyslipidemia) [314] insulin resistance [309,310,312], diabetes [315], obesity [309,311], elevated C-reactive protein [316], waist-to-hip ratio [309], reduced coronary flow reserve [279], smoking [317], sedentary life [318] and psychological distress [319], which act via effects on endothelial dysfunction, apoptosis and impaired endothelial-dependent vascular reactivity, have been associated with low serum IGF-I levels and reduced IGF-I and IGF-IR mRNA as well as protein expression in vascular smooth muscle cells (VSMC) [313]. Initially described as a pro-atherogenic molecule due to its proliferative role in VSMC, it was further demonstrated that the effect of IGF-I on VSMC was to compensate for local apoptosis, and that, overall, IGF-I is not pro-atherogenic in native arteries but anti-atherogenic [320], mainly through enhanced nitric oxide production [308]. In addition, IGF-I induces vasodilatation [279,321,322], thereby influencing the regulation of vascular tone and arterial blood pressure and preserving coronary flow reserve [279,321], platelet function, and glucose uptake [323].

Finally, it was recently suggested that IGF-I has significant characteristics to be a good marker for the insulin resistance syndrome and risk of cardiovascular disease, since logistic regression analysis showed that each unit increase in log-transformed IGF-I concentrations was associated with a 90.5% reduction in the risk of metabolic syndrome [274].

#### Neurodegenerative diseases

Neurodegenerative diseases are a heterogeneous group of disorders from virtually unknown etiology, which eventually lead to neuronal degeneration and dysfunction. As described before, since GH/IGF-I axis is involved in many aspects of brain development, growth and function, their progressive decrease during aging could be involved in a variety of human cerebrovascular diseases, comprising Alzheimer’s disease (AD), vascular dementia (VD), amyotrophic lateral sclerosis (ALS) and stroke.

Alzheimer’s disease and vascular dementia are the most common forms of dementia in elderly [324]. A decrease in IGF-I levels in AD and VD has been widely documented and it may be involved in the development of neurofibrillary tangles, abnormal amyloid β metabolism and aberrant Tau phosphorylation, cognitive loss, neural inflammation, oxidative stress or mitochondrial dysfunction, among others [68,325].

Specifically, IGF-I has been proposed as a physiological regulator of brain amyloid levels [68] by the entrance/production through the brain barrier of different proteins involved in Aβ transport, which eventually enhances its brain efflux [68,326]. In fact, blockade of systemic IGF-I action at the choroid plexus was sufficient to induce brain amyloidosis [326].

Another well recognised neuropathological finding in AD is the accumulation of abnormally hyperphosphorylated Tau in degenerating neurons [325]. Based on the known role of insulin/IGF-I as inhibitors of Tau phosphorylation by inhibiting a major Tau kinase, such as glycogen synthase kinase-β, numerous observations demonstrated that IGF-I controls the levels of hyperphosphorylated Tau in brain [326,327]. Two other pathological processes underlying neuronal decline in AD are gaining attention: oxidative stress and inflammation [328,329]. Again, antiinflammatory and antioxidant effects of IGF-I, together with its antiapoptotic capability, endorse IGF-I as a suitable candidate for AD treatment. Thus, although more work in animal models are required, the available evidence strongly indicates that IGF-I therapy in Alzheimer’s dementia may address etiopathogenic processes and could be a potential candidate for clinical trials.

On the other hand, amyotrophic lateral sclerosis is the most common motor neuron disorder in human adults. In this pathology, IGF-I levels diverge between studies, from increased to decreased IGF-I concentrations [330-332], probably due to differences in samples origin (serum/cerebrospinal fluid vs spinal cord/musculoskeletal) and could be explained as a physiological defensive mechanism promoted in response to the neural degeneration and/or muscle atrophy. Beneficial effects of IGF-I treatment in ALS have been demonstrated both *in vivo* and *in vitro*, from which IGF-I has been postulated as an important factor for the maintenance and survival of motor neurons in the spinal cord by activating clue pathways as PI3K/Akt and p44/42 MAPK [333] and ameliorating the superoxide effect [334]. Based on the strength of the pre-clinical evidence, two randomized double-blind placebo-controlled phase III trials examining the efficacy of subcutaneous rhIGF-I in the treatment of ALS have been completed [335,336]; however, the results of these two trials conflict, concluding, that more complex studies to set the potential role of IGF-I in ALS disease are required.

Along with this, cerebrovascular accident (CVA) is currently the second leading cause of death in the Western world, ranking after heart disease and before cancer [337], and experts predict that it is likely to be soon the most common cause of death worldwide [338]. Studies on patients with ischemic stroke suggest that high circulating IGF-I levels are associated with neurological recovery and a better functional outcome [339], probably due to its neuroprotective and pro-angiogenic effects. In fact, increased cerebrovacular mortality in patients with hypopituitarism was documented last century [340]. Importantly, as the nervous system ages, there is also a rarefaction of the microvasculature in different regions of the brain, as well as alterations in the structure of the remaining vessels, which have been causally linked to cognitive dysfunction in older humans [341,342]. This age-related microvascular rarefaction contributes to a decline in regional cerebral blood flow that reduces metabolic support for neural signaling, especially when neuronal activity is high. Of great interest, infusion of IGF-I was shown to elicit a significant (~40%) increase in cerebromicrovascular density in the adult mouse brain [343], via stimulation of HIF-1α and its downstream effector, VEGF.

Menupause and insulin resistance have independently been involved in the incidence of neurodegenerative diseases [344]. Several studies have pointed to PI3K activation as a pivotal event for estradiol effects, a common pathway for IGF-I and insulin [344]. Therefore, it seems possible that estrogen receptor alpha may interact with IGF-I/insulin signaling pathways, to promote neuroprotective effects in brain. Current investigations are also evidencing the role exerted by other key signalling molecules, such as glycogen synthase kinase 3 and beta-catenin, in the cross-talk of estrogen receptors and IGF-I receptors in neural cells [345].

Cognition and memory also decline with age, and they have been correlated to low IGF-I levels as well [346-349], through a proposed mechanism that may include its role in angiogenesis and neurogenesis in the hippocampus. Interestingly, the phosphodiesterase inhibitor Cilostazol (used in peripheral vascular disease treatment) is able to improve cognitive function in mice by increasing the hippocampal production of IGF-I through stimulation of sensory neurons [350].

#### Musculoskeletal disorders

Aging is associated with a decline in skeletal muscle mass, sometimes referred to as “sarcopenia of old age”. There are several underlying mechanisms that have been implicated in this age-related muscle wasting: decreased protein synthesis, reduced enzymatic activity (especially in glycolytic and glycogenolytic pathways), depletion in energy reserves, increased oxidative damage, and changes in ion content [351], among others. GH and IGF-I have a significant anabolic effect on skeletal muscle and so their decline with aging likely contributes to the decline in muscle mass. For example, they can promote mitosis, protein synthesis, satellite cell proliferation and nerve sprouting, while preventing apoptosis [352,353]. However, in general, tissue responsiveness to IGF-I is altered with aging. Thus, it was reported that median IGF-I and IGFBP-5 mRNA levels in resting young muscle are more than twice higher than those in elderly muscle [354], and aging is associated with decreases in IGF-IR content and IGF-IR phosphorylation in muscle [355].

Skeletal health may also be compromised in vertebrates with reduced GH/IGF-I signaling. Like muscle, normal aging is associated with both quantitative and qualitative changes in bone, including alterations in trabecular architecture, mineralization, protein content and the accumulations of microfractures [356]. IGF-I, which mediates most of the effects of GH on skeletal metabolism, promotes chondrogenesis and increases bone formation by regulating the functions of the differentiated osteoblasts [357,358]. Furthermore, in fibroblasts, DNA synthesis and cell proliferation in response to IGF-I decrease with elderly [359]. Bone responsiveness to IGF-I also decreases with aging, requiring higher doses to reach the same anabolic effect [360]. These studies suggest that low circulating IGF-I bioactivity and/or abnormalities of IGF-I signaling in elderly subjects, may play an important role in age-related sarcopenia and osteopenia, where the substitutive IGF-I treatment may be a suitable therapeutic strategy, although this apparent IGF-I resistance may dampen this aim.

### Other IGF-I deficiency conditions

#### Renal diseases

As previously stated, the IGF system is involved in normal kidney development, and age-related dysregulation of this system may play a role in kidney and vascular diseases, including hypertension [138]. In addition, under renal dysfunction conditions there are profound changes in renal responses to GH/IGF-I system as well as in the circulating levels of these hormones, despite the limited role of the kidney for removing IGF-I from the circulation (since negligible amounts of the IGFBP-3/ALS/IGF-I ternary complex crosses the glomerular barrier in healthy individuals) [361].

In mice with global inactivation of the IGF-I gene, the proportionally reduced kidney size associates with reduced glomerular size and decreased numbers of nephrons [362]. In addition, IGF-I has rapid effects on renal hemodynamics, including an increased renal blood flow and glomerular filtration rate [138,141,142]. Furthermore, both IGF-I and the IGF-IR are expressed in the developing glomerulus and patterns of expression are disrupted in animal models and in human examples of renal disease [140]. Indeed, it has been demonstrated a role of IGF-I signaling for maintaining glomerular integrity, restoring podocyte abnormalities, inhibiting podocyte apoptosis, and alterations in the glomerular basement membrane and the adjacent endothelial cell layer [140,363].

In humans, IGF-I also increases renal blood flow and GFR by ~25% and causes volume expansion and sodium retention by a direct action on the renal tubules, with stimulation of rennin release and suppression of atrial natriuretic peptide secretion [364]. IGF-I administered to GH deficient rats (or to patients with GH receptor defects) normalizes the low GFR as does GH replacement in GH deficiency. Curiously, the effects of GH on kidney function are similar to those observed with IGF-I, except that the functional response to GH is delayed several days, correlating with the secondary increase in serum IGF-I levels, and thus indicating that the GH effects are mediated by IGF-I. However, it is noteworthy that GH receptors are present in the proximal tubule, a site where IGF-I mRNA is not normally expressed, suggesting that GH also may have direct actions on tubular function [364].

In another hand, while most reports appear to implicate IGF-I as a potential mediator of pathological changes in the diabetic kidney [364,365], IGF-I is also protective against oxidative stress and apoptosis induced by high levels of glucose in cultured mesangial cells. This protection appears to be mediated by Akt/PKB and MAPK signalling pathways [366], and it has been suggested that stimulation of this survival pathways may be turned to therapeutic advantage for protection against cell death and progression of nephropathy.

#### Catabolic states

Clinical investigators have shown that IGF-I levels are often significantly altered in catabolic states, including the acute postoperative period [367], burn patients [368] and chronic catabolic illnesses, such as cystic fibrosis [369] and HIV with wasting [370]. These conditions result in low IGF-I concentrations, and changes in IGF-I positively correlate with changes in lean body mass [369,370], as well as reversal of acute catabolic states is associated with an increase in IGF-I levels [371,372]. In clinical use, children with extensive thermal burns who were treated with IGF-I in combination with IGFBP-3, presented a reduction in serum levels of IL-1β, TNF-α, C-reactive protein, α1-acid glycoprotein, and complement C-3 [299]. In contrast, the serum levels of retinol-binding proteins, prealbumin, and transferrin were increased by the infusion. From these results, authors concluded that attenuating the pro-inflammatory acute phase with IGF-I/IGFBP-3 may prevent multiple organ failure and improve clinical outcomes after thermal injury without any detectable adverse side effects.

Also, when IGF-I was used to monitor total parenteral nutrition therapy in catabolic patients, the changes correlate with improvements in protein metabolism [373]. Consistently, a close correlation between IGF-I and protein synthesis in patients with burns was reported [368]. Similarly, extremely low IGF-I levels observed in severe malnutrition improved with caloric repletion [374].

## Current therapeutic options and limitations

The pluripotential roles of IGF-I may explain the interest and wide availability of IGF-I assays from different laboratories. However, standard methods of IGF-I measurement are not well characterized yet. Recombinant human IGF-I was first available for experimental therapy in the late 1980s, what allowed the development of long-term studies in children with severe primary IGF-I deficiency (defined as a height SDS and IGF-I SDS less than or equal to −3 and normal or elevated GH levels) [375]. These studies followed the children for up to 12 years and reported a significant dose-dependent increase in mean first-year height velocity over baseline (~3.0 cm/year at baseline, ~8.5 cm/year at first year, p<0.001) [376-380]. Mean height velocity decreased during the subsequent years of treatment, but remained higher than the pretreatment height velocity for up to 8 years [376]. A complementary aspect of the rhIGF-I treatment in these patients was an increase in testosterone levels, testicular size and stretched penile length, which indicates a direct effect of IGF-I on sex hormones and organs in male patients [381].

These studies supported the 2005 US Food and Drug Administration (FDA) approval of rhIGF-I, being commercially available in 2005 for treatment of patients with severe primary IGF-I deficiency due to genetic GH resistance from mutations in the GH receptor, defects in the GH receptor signaling pathway (including STAT5b gene mutations), mutations in the IGF-I gene, or in rare patients with GH gene deletions in whom inactivating antibodies develop to exogenous rhGH [382-384], but not for other conditions of (secondary) IGF-I deficiency such as malnutrition, hypothyroidism and chronic illnesses [383]:

• Active or suspected neoplasia.

• Allergy to rhIGF-I (mecasermin, Increlex®) or any of its ingredients.

• Chronic illness (i.e. diabetes, cystic fibrosis, etc.).

• Growth failure associated with other identifiable causes (i.e. Prader-Willi syndrome, Russell-Silver syndrome, Turner syndrome, Noonan syndrome or chromosomal abnormalities).

• Patients with closed epiphyses (for promoting growth treatments).

By the same time, FDA also approved the use of the combination of IGF-I and IGFBP-3. Theoretically, it might be a better choice as a more physiological strategy (which would require lower doses). However, the results were not as promising as expected and legal issues led to its discontinuation. Interestingly, a recent work developed in two brothers with insulin-like growth factor deficiency, where one was treated with IGF-I plus IGFBP-3 and the other with just IGF-I, showed that both modalities improved determinants of hepatic insulin sensitivity, body composition and linear growth rate; however, IGF-I alone seemed to be more efficient [385].

A complete review by Laron in 2008 [386] summarizes the experience by several groups worldwide. The main conclusions were: 1) The one or two injections regimen result in the same growth velocity; 2) The growth speed obtained with IGF-I administration is smaller than that observed with hGH in children with congenital isolated GH deficiency; 3) Overdosage of IGF-I causes a series of adverse effects which can be avoided by carefully monitoring the serum IGF-I and GH levels. The optimal dosing guidelines are still on debate. Firstly stated on the basis of IGF-I tolerance, two more recent studies presented at the International Congress of Endocrinology (2008) showed the safety and efficacy of both twice-daily (80–120 μg/Kg) or once-daily (240 μg/Kg) rhIGF-I treatment for primary IGF-I deficiency [387,388]. Interestingly, it was also reported a certain degree of heterogeneity in the serum IGF-I responses, probably influenced on the IGFBP-3 levels [389].

Another disturbing aspects are the difficulties found when collecting, storing and monitoring IGF-I serum samples [389,390]. In contrast to GH, circulating levels of IGF-I remain relatively stable during daytime with minimum oscillations, being unaffected by meal intake [391,392]. However, IGFBP-3 levels showed acute changes with meal intake [391-393], what has direct consequences on IGF-I bioavailability, and it was reported a nocturnal decline of IGF-I levels from midnight to 4 am [393], probably due to shifts in plasma volume. Despite this, the lack of any major diurnal variation in circulating IGF-I levels, combined with the long half-life of ternary complex constituting IGF-I [393] has led to the concept that a single measurement of IGF-I is representative for an individual IGF-I level. On the other hand, the serum half-life of rhIGF-I is less than 24 hours, what suggests that serum IGF-I monitoring may be worth for detecting a single missing dose on the day, but it is not useful for identifying long term treatment noncompliance [394]. Furthermore, there is no clear indication to date that routine serum IGF-I monitoring is informative or useful for children taking rhIGF-I treatment, as it has not been linked to the occurrence of adverse events or efficacy outcomes [387-389].

Safety is the paramount aim when developing new drugs. In this case, as previously happened with recombinant technologies for GH and insulin, IGF-I is a natural peptide already produced in humans, what facilitates the elucidation of possible secondary effects due to rhIGF-I therapy. However, a number of clinical trials monitoring long-term rhIGF-I treatment have reported different adverse events [372,377,387-389,395-398], although they were transient, well tolerated and easy managed without treatment discontinuation (Table 1). In brief, it has been reported episodes of tachycardia (self-resolved and probably due to the inotropic effect of rhIGF-I), transient increase of intracranial pressure with headache and vomiting (consistent with the safety profile of rhGH treatment), lipohypertrophy at injection site, tonsillar/adenoidal hypertrophy, facial edema, arthralgia, myalgia, asthenia, orthostatic hypotension and hypoglycemia. This decrease in blood sugar could be a consequence of the insulin like action of IGF-I *per se*, and the binding of IGF-I to the insulin receptor. However, a placebo controlled study by Guevara-Aguirre et al. in 1995 reported no statistically significant difference in the frequency of hypoglycemia in those who received IGF-I vs. placebo for 6 months (86% in IGF-I group vs. 67% in the placebo group) [379]. Interestingly, as previously stated by Laron [386], doses lower than 60 μg/Kg/day did not show these described adverse effects, both in human clinical trials [398,399] and in animal experimental models [55,56,219-221,223].

**Table 1:** Reported adverse effect during IGF-I treatment

**Adverse effect**	**IGF-I doses (μg/Kg/day)**	**References**
Tachycardia	150-200	[397]
	120-180	[398]
Hypoglycemia	60-120	[377,380]
	80-120	[378,387]
Intracrenial pressure increment	80-120	[378,387]
Lipohypertrophy	60-120	[377,387]
Tonsillar/adenoidal hypertrophy	60-120	[377,387]
Headache and vomiting	80-120	[387]
Facial edema	120-180	[398]
	120	[373]
Asthenia, orthostatic hypotension	120	[373]
Arthralgia/myalgia	120	[373]

Subcutaneously, daily, for at least 1 year.

Another outcome from the rhIGF-I treatment in Laron syndrome is the significant increase in body adipose tissue (to double or triple the normal values) concomitantly with the increase in growth [400] although, due to the underdevelopment of the muscular and skeletal systems, body mass index did not accurately reflect the degree of obesity. From these data, authors concluded that IGF-I, similar to insulin, exerts an adipogenic effect. However, another study in insulin resistance, reported a reduction in both intramyocellular lipids and intrahepatic fat in a patient treated with rhIGF-I/IGFBP-3 (together with a post-treatment normalization of % HbA1C value) [401]. These data, although very preliminary, may indicate different mechanistic pathways by which IGF-I controls lipid metabolism.

Consistently, despite these known effects, we still must be alert due to the reported potential role of IGF-I in neoplasias development (a recommended review is available at ref. [402]). Fortunately, a recent study shed some light on this topic by reporting the malignancies occurrence in patients with congenital IGF-I deficiency and insensitivity to GH. They found that this condition seemed to be protective against future cancer development, but more interesting, even when treated with IGF-I [403]. Furthermore, no current data in the literature suggest the malignant transformation of a normal cell in association with IGF-I administration, what may reassure possible concerns about its long-term safety and use in replacement therapies.

Nowadays, rhIGF-I therapy is also being tested or proposed in other pathological conditions as chronic liver disease, insulin resistances/hyperinsulinemia, diabetes, neurological disorders (Alzheimer’s disease and amyotrophic lateral sclerosis) and stroke, cysticfibrosis, wound healing, AIDS muscle wasting and HIV-associated adipose redistribution syndrome, burns, osteoporosis, Crohn’s disease, Werner syndrome, X-linked severe combined immunodeficiency, myotonic muscular dystrophy, hearing loss prevention, spinal cord injury, cardiovascular protection, anorexia nervosa, fetal growth restriction and prevention of retinopathy of prematurity [182,303-306,384,399,401,404-423], although some of the treatment’s contraindications may limit its potential applicability (Table 2).

**Table 2:** Current proposed IGF-I treatments

**Pathological condition**	**References**
Laron Syndrome	[33-35,200-208,381-389,397]
Liver cirrhosis	[219-234,399]
Aging	[55,56,169,245-248,384]
IUGR	[37-41,182-197]
Neurological disorders	[335,336,405-407]
Stroke	[337,413]
Spinal cord injury	[421]
Cardiovascular protection	[270-277,310,415]
Diabetes	[302-306]
Insulin resistances/hyperinsulinism	[55,404,409,423]
Metabolic syndrome	[301,316]
Osteoporosis	[416,417]
Cystic fibrosis	[408]
Wound healing	[410]
Myotonic dystrophy	[419]
AIDS muscle wasting	[411]
HIV-associated adipose redistribution syndrome	[411]
Burns	[299]
Crohn’s disease	[412]
Werner syndrome	[417]
X-linked severe combined immunodeficiency	[418]
Hearing loss prevention	[414]
Anorexia nervosa	[420]
Retinopathy of prematurity	[422]

rhIGF-I replacement therapy may be recommended only to restore IGF-I deficiency conditions. In consequence, despite the number of proposed pathological conditions that may benefit from rhIGF-I treatment, up to date, only those in the white have been well characterized. Conditions in clear grey are supported by a wide body of evidence which render these circunstances prone to be classified as the first group. Dark grey are conditions that may need more research in order to adequately propose the IGF-I treatment as a proper strategy.

Nonetheless, in spite of the great amount of data obtained from these studies, there is still a necessity for further studies to either elucidate the right doses to achieve the expected results, clarify the rhIGF-I therapy effectiveness or even exclude any potential adverse event. For example, the effect of rhIGF-I in chronic liver injury (20 μg/Kg/day with dose escalation up to 50–100 μg/Kg/day for 4 months) was mainly reduced to an increase in albumin levels [399], probably due to the low number of subjects and treatment guidelines, which could not achieve normal IGF-I values, as was also reported in a recent study [387] where doses by 40 μg/Kg/day did were not able to normalize serum IGF-I.

In our experience, the most important goal is to prove the presence of IGF-I deficiency. However, in some cases like compensated liver cirrhosis, IGF-I circulating levels are normal, but if low doses of this hormone are administrated, many beneficial effects are induced [219-221,223,225,228,232]. This finding suggests that the bioavailability of IGF-I is reduced from early stages of liver disease. However, without hepatopathy, IGF-I levels and bioavailability are not diminished. Thus, IGF-I treatment failed to induce the desired effects, like was reported in a model of testicular dysfunction secondary to epinephrine intra-scrotal injections (but without liver cirrhosis or any other condition of IGF-I deficiency) [424].

At this point, it is also crucial to state that testicular dysfunction was only restored when it was associated to IGF-I deficiency conditions, since IGF-I treatment of rats with testicular damage secondary to epinephrine intra-scrotal injections (but without liver cirrhosis or any other condition of IGF-I deficiency) was not able to adequately recover their morphology and function.

On the other side, therapeutic use of rhIGF-I has been debated in ALS, since a Cochrane systematic evidence review found that the available randomized placebo controlled trials [335,336] did not permit a definitive assessment of its clinical efficacy [407], with just a small significant benefit in favor of recombinant IGF-I, which clinical relevance is unclear. The authors concluded that more research is needed, noting that one trial is in progress, and they recommended that future trials may include survival rates as another outcome measure.

And thirdly, although Rosenbloom [384] stated that the rhIGF-I insulin-sensitizing effect may be beneficial for diabetic patients to reduce hemoglobin A1C levels, there are no current ongoing clinical trials because of concern about risk of retinopathy and other complications [425], thus limiting the theoretically wide spectrum of potential IGF-I treatment indications.

## Conclusions

Briefly, IGF-I deficiency states –as it is the case with every other hormone- produce effects that culminate in recognizable syndromes with significant clinical consequences. Until now, the best known conditions of IGF-I deficiency are: Laron Syndrome, in children; advanced liver cirrhosis, in adults; and aging including cardiovascular and neurological diseases associated to aging. More recently, intrauterine growth restriction seems to appear as another state of IGF-I deficiency. In these four conditions, the replacement therapy can logically induce beneficial actions. Apart from these, many other diseases have been recently proposed to be either consequence or cause of both a systemic or partial IGF-I deficiency. However, more in-depth studies are needed to properly characterize these potential new conditions of IGF-I deficiency and their future clinical perspectives.

On the other hand, the multiple physiological properties of IGF-I may generate excessive prospects if basic concepts about this topic are not adequately considered:

1. IGF-I is a closed regulated hormone. Its therapeutic applications should, in principle, be limited to restore physiological levels, but not over its normal range, as it is the usual practice in endocrinology diseases (hypothyroidism, diabetes, etc.).

2. Exogenous administration of IGF-I in conditions without IGF-I deficiency, usually responds to an attempt to exploit its anti-inflammatory, hematopoietic, antioxidant, metabolic or anabolic properties. However, despite the limited results of these strategies, it may entail obvious risks. In the best of cases, these strategies would require prudence and short periods of treatment, as in the corticotherapy.

3. Low doses of IGF-I seem to be able to restore circulating levels of this hormone promoting beneficial effects without secondary effects (including hypoglycemia). Secondary adverse effects from IGF-I therapy were reported after administration of doses higher than 60–80 μg/Kg/day.

4. The axis GH/IGF-I is claiming a particular physiological understanding. Usually IGF-I deficiency is associated to “GH resistance” or “GH insensibility” states. IGF-I replacement therapy induced a restoration of the altered GH/IGF-I axis by reducing circulating GH levels and improving somatostatinergic tone. Thus, IGF-I therapy would need to be considered as a different strategy from GH treatment, avoiding past inadequate procedures when both GH/IGF-I therapies were considered equivalent.

5. Finally, a concern about the potential relationship between IGF-I and cancer is an awkward issue that may require a deeper approach. For instance, a question may arise: are the higher circulating IGF-I levels a marker or a causal factor? In our experience, IGF-I at low doses is a cytoprotective factor that, exerting effective mitochondrial protection, anti-inflammatory and antioxidant activities, could prevent oncogenesis and cancer development by acting in the earliest stages of these pathogenic mechanisms. In addition, despite permanent reservations relating cancer and IGF-I, no current data suggest an IGF-I related malignant transformation from a normal cell.

In conclusion, on the basis of the available evidence, and despite the current limitations in this matter, it seems reasonable that IGF-I therapies may be designed only to restore its physiological levels as a replacement treatment, but never elevating IGF-I levels above its upper normal range.

## Abbreviations

ALS: Acid-labile subunit; Aβ: Amyloid β; BBB: Blood–brain barrier; CVA: Cerebrovascular accident; EGF: Epidermal growth factor; CNS: Central nervous system; CVD: Cardiovascular diseases; EAE: Experimental autoimmune encephalomyelitis; FDA: Food and drug administration; FSH: Follicle-stimulating hormone; GFR: Glomerular filtration rate; GH: Growth hormone; GHBP: Growth hormone binding protein; GHI: Growth Hormone Insensitivity; HGF: Hepatocyte growth factor; HIF: Hypoxia inducible factor; HPA: Hypothalamo-pituitary-adrenal; IGF: Insulin-like growth factor; *IGF*-*IR*: Receptor de IGF-I; IGFBP: Insulin-like growth factor binding protein; IL: Interleukin; IUGR: Intrauterine growth restriction; LH: Luteinizing hormone; LS: Laron syndrome; MAPK: Mitogen-activated protein kinase; MDA: Malon-dialdehyde; NO: Nitric oxide; NSILA: Non-suppressible insulin-like activity; NSC: Neural stem cells; PCC: Protein carbonyl content; PI3K: Phosphatidyl-inositol-3-kinase; Rh: Recombinant human; ROS: Reactive oxygen species; SDS: Standard deviation score; SH2: Src homology 2; *TNF*-*α*: Tumor necrosis factor-alpha; VEGF: Vascular endothelial growth factor; VSMC: Vascular smooth muscle cells.

## Competing interests

Authors have nothing to disclose.

## Authors’ contributions

JEP participated in compiling data and elaborating the manuscript. ICC critically revised the intellectual content of the review, and gave the final approval. All authors read and approved the final manuscript.
